# Decisions, outcomes, and learning from what didn’t go wrong

**DOI:** 10.2478/jccm-2026-0019

**Published:** 2026-01-30

**Authors:** Razvan Azamfirei

**Affiliations:** Department of Anesthesiology and Critical Care, University of Pennsylvania Perelman School of Medicine, Philadelphia, USA

We learn from outcomes, yet outcomes are unreliable teachers. In critical care, decisions are made with incomplete information, under time pressure, within systems that normalize workarounds, and where causality is opaque [1]. The feedback we receive later, whether a patient survives or dies or the extent of their recovery, reflects more than the decision itself. Physiology, system redundancies, timely intervention by a colleague, stochastic variance: all shape outcomes independently of our reasoning [2]. If we treat outcomes as verdicts on decision quality, we will systematically mislearn.

When outcomes are bad, the reflex is often to externalize causality [3]. Sometimes this is accurate. Patients arrive in extremis. Pathophysiology is unforgiving. But “nothing could have been done” closes the learning loop prematurely. Even when the final outcome was not modifiable, the care surrounding it often was: how quickly we recognized a change in trajectory, how often our interventions added burden without benefit, and how clearly we communicated prognosis to the family. These remain within our control and merit examination. The question that moves teams forward is not “What would you have done differently?” but “What will you do differently for the next patient?” The former invites untestable counterfactuals and can become recriminatory; the latter directs attention toward actionable change. The risk, of course, is that reflection becomes self-punishment, which impairs future care rather than improving it [4].

The less-examined problem is good outcomes. We rarely interrogate what succeeded [5]. The patient survived, the team moves on, and whatever errors occurred remain invisible, not because they didn’t happen, but because they didn’t declare themselves in harm. Learning from success requires asking what went right, what conditions made it possible, and whether those conditions were skill or circumstance [6]. This interrogation is harder than it sounds, which is precisely why it doesn’t happen.

Decision quality and outcome quality are not the same thing, but we routinely conflate them [7]. A sound decision can lead to a bad outcome; an unsound decision can lead to a good one. The dangerous case is the latter. When a questionable choice works out, nothing prompts us to examine it. The delay that the patient tolerated, the finding we missed that didn’t matter, the shortcut that succeeded. These build false confidence and embed risk into normal practice [8]. They deserve examination precisely because nothing signals that they need it.

Critical care will always be practiced under uncertainty. Outcomes will remain noisy, and we cannot change that. What we can change is our interpretation of them. Bad outcomes should prompt reflection without collapsing into blame. Good outcomes should prompt the same reflection, but nothing forces us to do it. That asymmetry is the problem. We examine decisions only when harm makes examination unavoidable. The discipline is to examine decisions even when nothing forces us to.
